# In vitro efficiency of Amikacin Inhale, a novel drug-device delivery system

**DOI:** 10.1186/cc12019

**Published:** 2013-03-19

**Authors:** N Kadrichu, S Boc, K Corkery, P Challoner

**Affiliations:** 1 Novartis Pharmaceuticals, San Carlos, CA, USA; 2 Nektar Therapeutics, San Francisco, CA, USA

## Introduction

**Figure 1:**
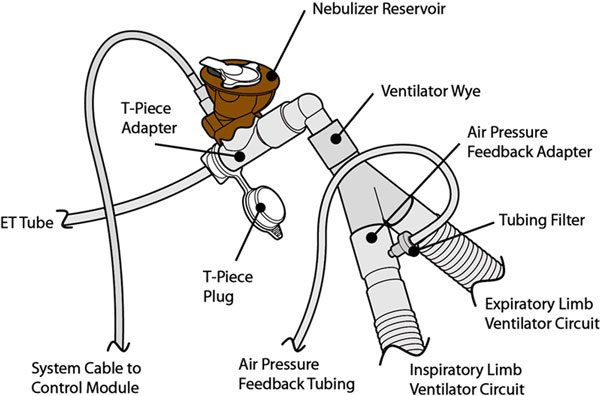
**On-vent system with brown blinder shell for clinical trials**.

**Figure 2:**
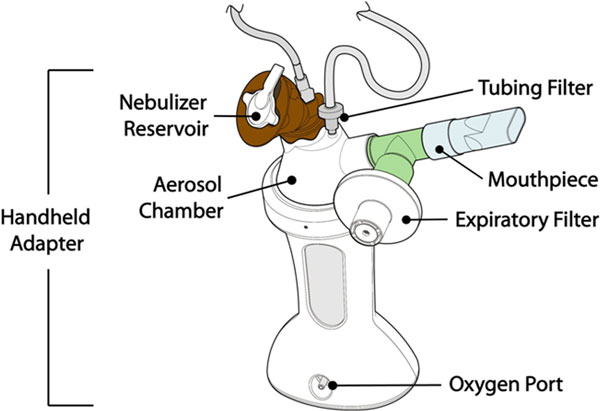
**Handheld system with brown blinder shell for clinical trials**.

Amikacin Inhale (NKTR-061, BAY41-6551) is a drug-device combination in clinical development for adjunctive treatment of intubated and mechanically ventilated patients with Gram-negative pneumonia. The product uses a proprietary vibrating mesh nebulizer system (PDDS Clinical) with amikacin sulfate formulated for inhalation (3.2 ml of 125 mg/ml amikacin solution) for a 10-day twice-daily course of therapy. It is designed for use with two delivery systems: one system for intubated patients (On-vent; Figure 1), and a second Handheld (HH) system for patients who are extubated before completing the course of therapy (Figure 2). We investigated *in vitro *the amikacin lung dose delivered by PDDS Clinical.

## Methods

An estimated lung dose (ELD) for On-vent setting was measured *in vitro *after collecting aerosolized amikacin from a filter at the end of an endotracheal tube during ventilation. The ELD for the HH device was calculated from the fine particle fraction (FPF <5 µm) post-mouthpiece, multiplied by the *in vitro *delivered dose post-mouthpiece. FPF <5 µm reflects lung deposition observed during phase 2 clinical trials [1]. Eighty-one nebulizers with volume median diameter (VMD) of 4.4 ± 0.5 µm and output rates of 0.23 ± 0.10 ml/minute were tested for each system. Delivered dose data were fit to the independent variables (that is, VMD and output rates) using a least-squares fit with 95% confidence limits.

## Results

Total percentage recoveries for On-vent and HH test runs were between 85% and 115% of the nominal dose. The mean ELDs were 50 ± 9% (On-vent) and 49 ± 11% (HH) of the nominal dose. Nebulizers with longer dosing times and lower VMDs had higher ELD values for both delivery systems.

## Conclusion

The results support the use of the PDDS Clinical with either system to administer aerosolized amikacin with high efficiency and no dose adjustment is required when switching from the On-vent to the HH system for extubated patients.
